# BRD4 promotes gouty arthritis through MDM2-mediated PPARγ degradation and pyroptosis

**DOI:** 10.1186/s10020-024-00831-w

**Published:** 2024-05-21

**Authors:** Xiaoxia Xu, Hongbin Qiu

**Affiliations:** Key Laboratory of Microecology-Immune Regulatory Network and Related Diseases, School of Basic Medicine, Jiamusi University, Jiamusi, Heilongjiang Province 154000 People’s Republic of China

**Keywords:** Gouty arthritis, Pyroptosis, BRD4, MDM2, PPARγ, Ubiquitination, NLRP3

## Abstract

**Background:**

Gouty arthritis (GA) is characterized by monosodium urate (MSU) crystal accumulation that instigates NLRP3-mediated pyroptosis; however, the underlying regulatory mechanisms have yet to be fully elucidated. The present research endeavors to elucidate the regulatory mechanisms underpinning this MSU-induced pyroptotic cascade in GA.

**Methods:**

J774 cells were exposed to lipopolysaccharide and MSU crystals to establish in vitro GA models, whereas C57BL/6 J male mice received MSU crystal injections to mimic in vivo GA conditions. Gene and protein expression levels were evaluated using real-time quantitative PCR, Western blotting, and immunohistochemical assays. Inflammatory markers were quantified via enzyme-linked immunosorbent assays. Pyroptosis was evaluated using immunofluorescence staining for caspase-1 and flow cytometry with caspase-1/propidium iodide staining. The interaction between MDM2 and PPARγ was analyzed through co-immunoprecipitation assays, whereas the interaction between BRD4 and the MDM2 promoter was examined using chromatin immunoprecipitation and dual-luciferase reporter assays. Mouse joint tissues were histopathologically evaluated using hematoxylin and eosin staining.

**Results:**

In GA, PPARγ was downregulated, whereas its overexpression mitigated NLRP3 inflammasome activation and pyroptosis. MDM2, which was upregulated in GA, destabilized PPARγ through the ubiquitin–proteasome degradation pathway, whereas its silencing attenuated NLRP3 activation by elevating PPARγ levels. Concurrently, BRD4 was elevated in GA and exacerbated NLRP3 activation and pyroptosis by transcriptionally upregulating MDM2, thereby promoting PPARγ degradation. In vivo experiments showed that BRD4 silencing ameliorated GA through this MDM2–PPARγ–pyroptosis axis.

**Conclusion:**

BRD4 promotes inflammation and pyroptosis in GA through MDM2-mediated PPARγ degradation, underscoring the therapeutic potential of targeting this pathway in GA management.

**Supplementary Information:**

The online version contains supplementary material available at 10.1186/s10020-024-00831-w.

## Introduction

Gouty arthritis (GA) is an inflammatory arthropathy precipitated by the deposition of monosodium urate (MSU) crystals into the joint tissues as a consequence of systemic hyperuricemia (Weaver et al. 2021). Patients with GA usually exhibit extreme pain, erythema, redness, and joint swelling (Liu et al. 2017). Failure to treated the joint promptly can cause stiffening and malformation, which might significantly increase their financial burden associated with treatment, impact their quality of life, and possibly lead to incapacity of action (Rees et al. 2014). Research has underscored the significance of pyroptosis in the pathophysiology of GA (Lin et al. 2020). Pyroptosis, a form of cell death characterized by inflammation, is dependent on caspase activation, typically triggered by inflammasomes such as NLRP3, resulting in membrane pore formation, cell lysis, and the release of cytokines (Ding et al. 2023; Huang et al. 2021). In the pathogenesis of GA, MSU crystals activate the NLRP3 inflammasome leading to caspase-1 activation, which consequently induces pyroptosis and proinflammatory cytokine release (Lin et al. 2020; Liu et al. 2023). This cascade amplifies joint inflammation, highlighting pyroptosis as a critical component and potential therapeutic target in GA. However, much mystery remains regarding the mechanism regulating NLRP3 inflammasome-mediated pyroptosis.

Peroxisome proliferator-activated receptor gamma (PPARγ), which belongs to the nuclear hormone receptor family, plays an important role in regulating various biological processes, including metabolism, inflammation, tumors, and immunity (Stark et al. 2021). PPARγ has emerged as a potential therapeutic target for improving GA (Wang et al. 2019; Wang and Jiang 2014a). In fact, Wang et al. revealed that PPARγ agonists demonstrated anti-inflammatory effects in GA by inhibiting the expression of TNF-α and IFN-γ (Wang and Jiang 2014b). In addition, PPARγ activation could inhibit GA progression by repressing the associated inflammation specifically induced by NLRP3 (Du et al. 2023). However, the mechanism by which PPARγ exerts its anti-inflammatory effects in GA remains unclear. Given that PPARγ can regulate NLRP3-mediated pyroptosis and inflammatory cytokine secretion (Li et al. 2023), one plausible hypothesis is that PPARγ may regulate NLRP3-associated pyroptosis, thereby influencing the course of GA. Hence, one of the objectives of the current study was to unravel this mystery.

Studies have confirmed that mouse models of GA induced by MSU crystals had elevated levels of PPARγ (Du et al. 2023); however, the molecular mechanisms underlying this aberrant upregulation of PPARγ remain unclear. Prior research has reported that PPARγ is subject to intricate post-transcriptional modulatory mechanisms (PTM), including ubiquitination (Li et al. 2016; Watanabe et al. 2015). However, the precise mechanisms regulating PPARγ ubiquitination within the context of GA have yet to be determined. The present investigation may elucidate the mechanism for PPARγ ubiquitination in GA and isolate the specific E3 ubiquitin ligase responsible for this modulation. Mouse double minute 2 (MDM2) is a suppressor of oncoprotein p53 (Yun et al. 2023). Notably, MDM2 is involved in regulating the development of various disease by acting as an E3 ubiquitin ligase. Indeed, one study showed that MDM2 promoted the progression of pulmonary arterial hypertension by mediating the ubiquitination of angiotensin-converting enzyme 2 (Shen et al. 2020). Moreover, previous reports have shown that MDM2 upregulation contributed to inflammation in atherosclerosis (Zeng et al. 2022). In the current study, we predicted that MDM2 could mediate ubiquitination degradation of PPARγ using Ubibrowser 2.0 prediction (http://ubibrowser.bio-it.cn/ubibrowser_v3/). However, whether MDM2 promotes NLRP3 inflammasome-mediated pyroptosis and the occurrence of GA by mediating the ubiquitination degradation of PPARγ still remains unknown, which deserves further research.

Recent studies have identified bromodomain-containing protein 4 (BRD4) as an essential molecule in GA. Notably, evidence has shown that BRD4 expression is upregulated in patients with GA (Shao et al. 2023) and that targeted inhibition of BRD4 can improve GA outcomes by inhibiting NLRP3-associated pyroptosis (Hao et al. 2020). Nevertheless, the specific mechanism by which BRD4 modulates pyroptosis in GA remains ambiguous. As a transcriptional factor, BRD4 has the potential to regulate pyroptosis through its transcriptional activity (Tan et al. 2020). Therefore, the current study sought to determine whether BRD4 regulates pyroptosis through its transcriptional activity and explore its interplay with PPARγ.

In this study, we investigated the function of pyroptosis in GA and elucidated the relationship between PPARγ and BRD4 during this process. Our findings revealed that BRD4 facilitated pyroptosis via the mouse double minute 2 homolog (MDM2)/PPARγ pathway, thereby contributing to the progression of GA.

## Methods

### Cell culture and treatment

J774 and 293 T cell lines were acquired from the American Type Culture Collection (ATCC; MD, United States). Cells were cultured in Dulbecco’s Modified Eagle Medium (Gibco, United States) containing 10% fetal bovine serum (Gibco, MD, United States) and 1% penicillin–streptomycin (Gibco) at 37 °C in a humidified atmosphere with 5% CO_2_.

For GA cell model experiments, J774 cells were seeded into a 6-well plate (1 × 10^6^ cells/well). Upon reaching 70%–80% confluence, they were treated with lipopolysaccharide (LPS, 100 ng/mL, Sigma-Aldrich, MO, United States) for 1 h to induce inflammation. Subsequently, cells were stimulated with MSU crystals (200 µg/mL, Invitrogen, CA, United States) for an additional 6 h.

For protein stability studies, cells were treated with MG132 (10 µM, Sigma-Aldrich) for 6 h or cycloheximide (100 µg/mL, Sigma-Aldrich) for 0, 15, 30, 60, 120, and 240 min. Protein levels were detected using Western blotting.

### Cell transfection

cDNAs encoding for PPARγ, BRD4, and MDM2 were individually cloned into the pcDNA3.1 expression vector (Youbio, Hunan, China) to create OE-PPARγ, OE-BRD4, and OE-MDM2 plasmids. Short hairpin RNA (shRNA) sequences targeting MDM2, TRIM25, HECW1, RBBP6, SIAH2, SIAH1, PPARγ, and BRD4 were individually cloned into pLKO.1 shRNA expression vectors (Youbio) to create sh-MDM2, sh-TRIM25, sh-HECW1, sh-RBBP6, sh-SIAH2, sh-SIAH1, sh-PPARγ, and sh-BRD4 plasmids.

Cells were cultured in 24-well plates (2 × 10^5^ cells/well) containing 500 µL of medium for 24 h and incubated with plasmids (0.5 µg/well) mixed with Lipofectamine 3000 (1.5 µL/well, Thermo Fisher Scientific, MA, United States) at 37 °C and 5% CO_2_ for 48 h. After the transfection period, the transfection efficiency was verified using Real-time quantitative PCR (RT-qPCR) or Western blotting.

### Animals and treatment

C57BL/6 J male mice (aged 8–10 weeks, weighing 20–25 g) were obtained from Hunan Slake Jingda Experimental Animal Center (Hunan, China). Experimental procedures were approved by Jiamusi University and conducted in accordance with national and international guidelines. Mice were anesthetized using 2% isoflurane (Sigma-Aldrich), after which a 30-gauge needle was used for the intra-articular knee injection of 50 µL of MSU crystal suspension (20 mg/mL in phosphate-buffered saline at pH 7.2; Invitrogen). The injection was performed bilaterally. Following MSU injection, another intra-articular knee injection of 50 µL of adenoviral sh-BRD4 was performed using a separate 30-gauge needle.

### Western blotting

Proteins from J774 cells were lysed using RIPA buffer (Thermo Fisher Scientific), supplemented with protease and phosphatase inhibitors (Roche, Basel, Switzerland). After centrifugation for 15 min of (14,000* g* at 4 °C), protein concentrations were determined using a BCA kit (Thermo Fisher Scientific). Protein (30 µg) was isolated using 10% SDS-PAGE and transferred to a PVDF membrane (Millipore, MA, United States), which was then blocked using 5% BSA (Sigma-Aldrich) for 1 h. The membranes were incubated with the primary antibodies overnight at 4 °C and subsequently incubated with the corresponding secondary antibodies (Abcam) for 1 h and washed. The bands were visualized using ECL reagents (Thermo Fisher Scientific) and imaged on a high-resolution system (Bio-Rad, CA, United States). Band intensities, normalized to β-actin (Abcam, Cambridge, UK), were quantified using software (Bio-Rad).

The primary antibodies used were as follows: anti-MDM2 antibody (Cell Signaling Technology, MA, United States), anti-TRIM25 antibody (Abcam), anti-HECW1 antibody (Sigma-Aldrich), anti-RBBP6 antibody (Thermo Fisher Scientific), anti-SIAH2 antibody (Santa Cruz Biotechnology, CA, United States), anti-SIAH1 antibody (Cell Signaling Technology), anti-NLRP3 antibody (Novus Biologicals, United States), anti-ASC antibody (Invitrogen), anti-GSDMD-N antibody (Abcam), anti-IL-1β antibody (BD Biosciences, NJ, United States), anti-c-caspase-1 antibody (R&D Systems, IL, United States), anti-BRD4 antibody (Cell Signaling Technology), anti-PPARγ antibody (Abcam), anti-Ubiquitin antibody (Abcam), and anti-IL-18 antibody (Santa Cruz Biotechnology). All antibodies were diluted according to the dilution recommended by the manufacturers’ instructions.

### RT-qPCR

Total RNA of the treated J774 cells was extracted using the Trizol reagent (Invitrogen). cDNA synthesis was performed using a reverse transcription kit (Thermo Fisher Scientific). Subsequently, PCR was conducted using SYBR Green Master Mix (Bio-Rad) on a real-time PCR system (Applied Biosystems, CA, United States). The primers used for MDM2 and BRD4 were as follows (sequences provided as 5′–3′): MDM2: forward, CTTCGTGAGAACTGGCTTCC; reverse, CTGTCAGCTTTTTGCCATCA; BRD4: forward, GCCTTTCAGCACCTCACTTC; reverse, GCTCCTGCTTCTGTTTGTCC. Each experiment was performed in triplicate. GAPDH was used to normalize the expression levels, which were calculated using the 2^(−ΔΔCt)^ method.

### Enzyme-linked immunosorbent assay (ELISA)

The cell supernatants were collected and centrifuged (2000* g* at 4 °C for 10 min) to remove cellular debris. Thereafter, the cleared supernatants were used for ELISA assays using commercially available ELISA kits for IL-1β and IL-18 (R&D Systems) following the manufacturer’s guidelines. The OD value was determined using a microplate reader (Thermo Fisher Scientific).

### Flow cytometry

To assess pyroptosis, caspase-1 activation and membrane integrity were measured using flow cytometry. Cells were trypsinized, washed with cold phosphate-buffered saline, and resuspended for staining. Caspase-1 activity was determined with FAM-YVAD-FMK using the FAM-FLICA Caspase-1 Assay Kit (ImmunoChemistry Technologies, CA, United States) for 1 h at room temperature. Membrane integrity was evaluated via a 5-min incubation with propidium iodide (PI; 5 μg/mL; Sigma-Aldrich) at 37 °C. Cells were then analyzed using a flow cytometer (BD Biosciences), and data were processed using FlowJo 7.6 (Treestar, OR, United States).

### Immunofluorescence

Cells were fixed with 4% paraformaldehyde (Sigma-Aldrich) for 15 min and permeabilized with 0.1% Triton X-100 (Sigma-Aldrich) for 10 min. After blocking for 1 h using 5% BSA (Sigma-Aldrich), cells were incubated with the primary anti-caspase-1 antibody (Cell Signaling Technology) overnight at 4 °C. After washing, the cells were exposed to Alexa Fluor-conjugated secondary antibody (Invitrogen) for 1 h. Nuclei were counterstained with DAPI (Cell Signaling Technology). Fluorescence was captured using a confocal microscope and imaged using Image J software (NIH, United States).

### Co-immunoprecipitation (Co-IP) assay

J774 cell lysates were prepared using IP lysis buffer (Thermo Fisher Scientific) supplemented with protease and phosphatase inhibitors (Roche). Protein A/G agarose beads (Santa Cruz Biotechnology) were added to the cell lysates for 1 h at 4 °C. Thereafter, precleared lysates were incubated with anti-MDM2 antibody (Cell Signaling Technology), TRIM25 (Abcam), RBBP6 (Thermo Fisher Scientific), SIAH2 (Thermo Fisher Scientific), or anti-PPARγ antibody (Abcam) overnight at 4 °C. The next day, Protein A/G Agarose was added to the sample, which was then incubated for 2 h at 4 °C. The immunoprecipitated complexes were eluted by boiling in 2 × Laemmli buffer. The eluted proteins were analyzed through Western blotting to detect MDM2, TRIM25, RBBP6, SIAH2, and PPARγ.

### Chromatin immunoprecipitation (ChIP) assay

J774 cells were cross-linked with 1% formaldehyde for 10 min (Sigma-Aldrich) and then quenched by 0.125 M glycine. Subsequently, cells were lysed and sonicated to shear chromatin to segments (200–500 bp). Segments were pre-cleared with Protein A/G Agarose beads (Invitrogen) and then incubated overnight at 4 °C with anti-BRD4 antibody (Cell Signaling Technology) or IgG. The next day, Protein A/G Agarose beads were added, after which the complexes were eluted and uncross-linked. Purified DNA was analyzed via PCR assay to assess the binding of BRD4 to the BS1–3 sites on the MDM2 gene.

### Dual-luciferase reporter assay

Wild-type (WT) or BRD4 binding site 1 (BS1)-mutated MDM2 promoter sequences were inserted into the pGL3-luciferase reporter vector. Cells were co-transfected with the established luciferase reporter plasmid and sh-NC or sh-BRD4 for 48 h. Luciferase activities were quantified using the Dual-Luciferase Reporter Assay System (Promega, WI, United States). Data were analyzed to determine the impact of BRD4 on MDM2 BS1 transcriptional activity.

### Hematoxylin and eosin (H&E) staining

Tissues were fixed in 10% neutral-buffered formalin (Sigma-Aldrich) for 24 h and embedded in paraffin wax (Thermo Fisher Scientific). Sections of 5 µm were then prepared using a precision microtome. Following deparaffinization in xylene (Thermo Fisher Scientific) and rehydration through graded ethanol solutions, the sections were stained with Gill’s hematoxylin (Thermo Fisher Scientific) for nuclear visualization and counterstained with eosin Y solution (Sigma-Aldrich) for cytoplasmic and extracellular matrix components. After a series of dehydration steps in graded ethanol and clearance in xylene, slides were mounted using Permount mounting medium (Thermo Fisher Scientific). Microscopic examination was subsequently conducted to assess histological features indicative of joint pathology.

### Immunohistochemical (IHC) assay

For IHC analysis of mouse tissues, 5-µm-thick formalin-fixed, paraffin-embedded sections were processed using citrate buffer (Sigma-Aldrich). Thereafter, 3% hydrogen peroxide (Sigma-Aldrich) was added to quench endogenous peroxidase activity. The sections were then blocked using 5% BSA (Sigma-Aldrich) and incubated with the primary antibodies for BRD4 (Cell Signaling Technology), MDM2 (Abcam), and PPARγ (Santa Cruz Biotechnology) overnight at 4 °C. Sections were then exposed to biotinylated secondary antibodies (Vector Laboratories, United States) and visualized using DAB (Sigma-Aldrich). Counterstaining was performed using hematoxylin (Thermo Fisher Scientific). Staining intensity and distribution were evaluated microscopically.

### Statistical analysis

All experiments were conducted in triplicate to ensure reproducibility. Statistical analyses were performed using GraphPad Prism 9.0 (GraphPad Software, United States). Student’s t-test was used for comparisons between two groups, whereas one-way ANOVA followed by Tukey’s post-hoc test was used for comparisons between multiple groups. Data were presented as means ± standard deviation (SD). A p value of less than 0.05 indicated statistical significance.

## Results

### PPARγ overexpression inhibited NLRP3 inflammasome activation and pyroptosis in the GA cell model

**Fig. 1:**
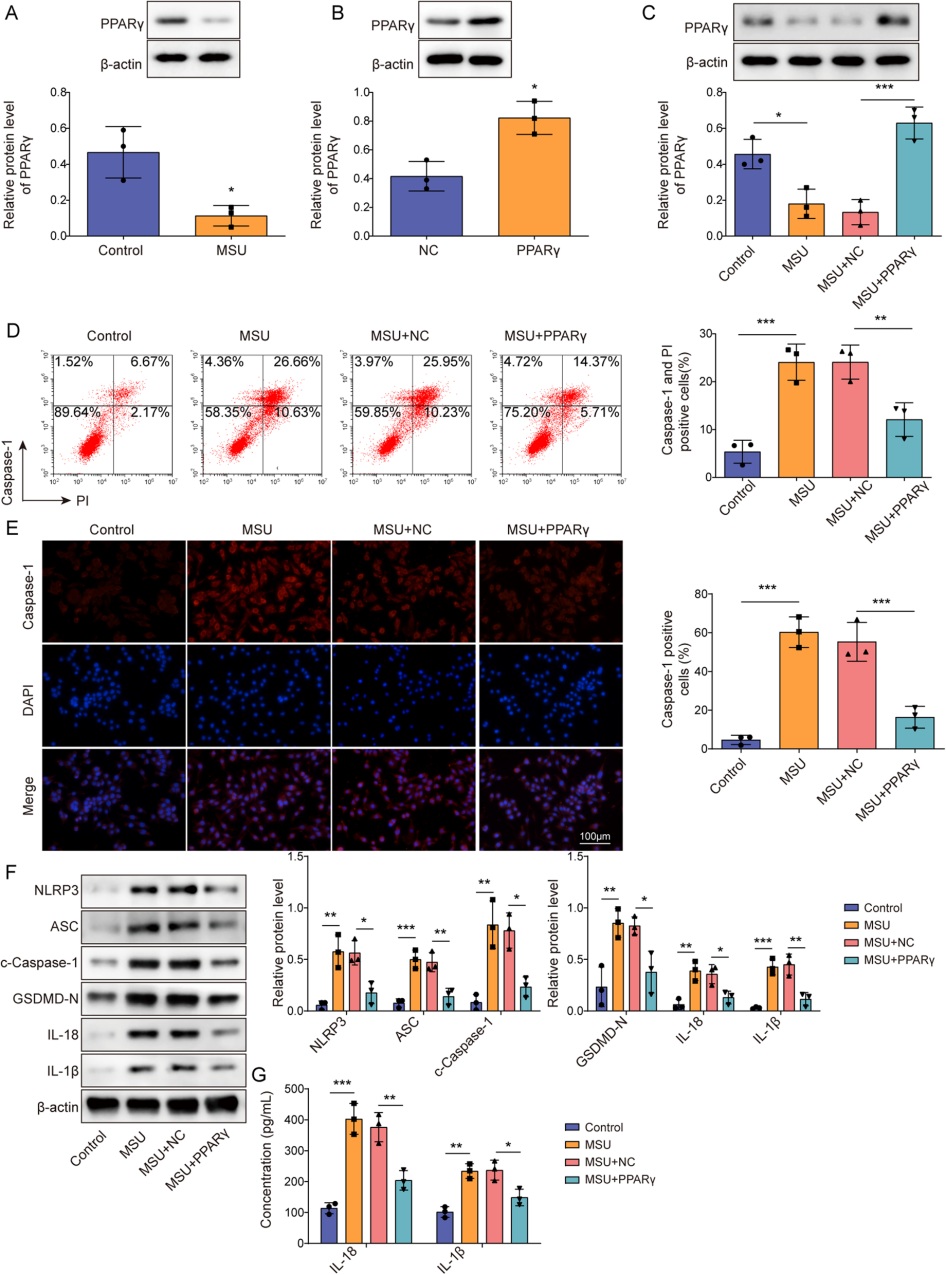
PPARγ overexpression inhibited NLRP3 inflammasome activation and pyroptosis in the GA cell model. **A** J774 cells were treated with LPS and MSU, after which PPARγ protein levels in the cells were determined via Western blotting. **B** PPARγ protein levels in J774 cells after NC or OE-PPARγ transfection was examined via Western blotting. J774 cells were treated with LPS and MSU and transfected with NC or OE-PPARγ. **C** PPARγ protein levels in J774 cells were determined using Western blotting. **D** Flow cytometry following caspase-1/PI staining was utilized to examine cell pyroptosis. **E** c-caspase-1 expression was detected by immunofluorescence (scale bar = 100 µm). **F** The levels of pyroptosis-related protein (NLRP3, ASC, c-caspase-1, GSDMD-N, IL-1β and IL-18) in J774 cells were assessed via Western blotting analysis. **G** ELISA was performed to measure IL-1β and IL-18 levels in cell supernatants. Measurement data are presented as mean ± SD. All data were obtained from three replicate experiments. *P < 0.05, **P < 0.01, ***P < 0.001 versus indicated group

To investigate the involvement of PPARγ in regulating inflammation during GA development, J774 cells were treated with LPS and MSU to generate a GA inflammation cell model. We initially observed that LPS and MSU markedly reduced PPARγ expression level in J774 cells (Fig. 1A). Subsequently, we determined whether this abnormality in PPARγ protein expression influenced NLRP3 inflammasome activation in GA pathology. Accordingly, we induced PPARγ overexpression in LPS- and MSU-treated J774 cells. Our results showed that OE-PPARγ transfection significantly elevated PPARγ protein level in J774 cells, with the transfection efficiency being presented in Fig. 1B. Our results also indicated that PPARγ overexpression reversed its downregulation caused by LPS and MSU (Fig. 1C). Subsequently, PPARγ overexpression attenuated LPS- and MSU-induced pyroptosis of J774 cells (Fig. 1D). Additionally, we also detected changes in pyroptosis-related molecules, with our results demonstrating that PPARγ overexpression reversed the upregulated expression of NLRP3, ASC, GSDMD-N, and c-caspase-1 proteins and elevated concentrations of IL-1β and IL-18 in GA cell model, indicating that PPARγ suppressed NLRP3 inflammasome activation (Fig. 1E–G). In conclusion, PPARγ overexpression exhibited an inhibitory role against NLRP3 activation and pyroptosis in the GA cell model.

### MDM2 reduced PPARγ protein stability via the ubiquitin–proteasome degradation pathway

**Fig. 2:**
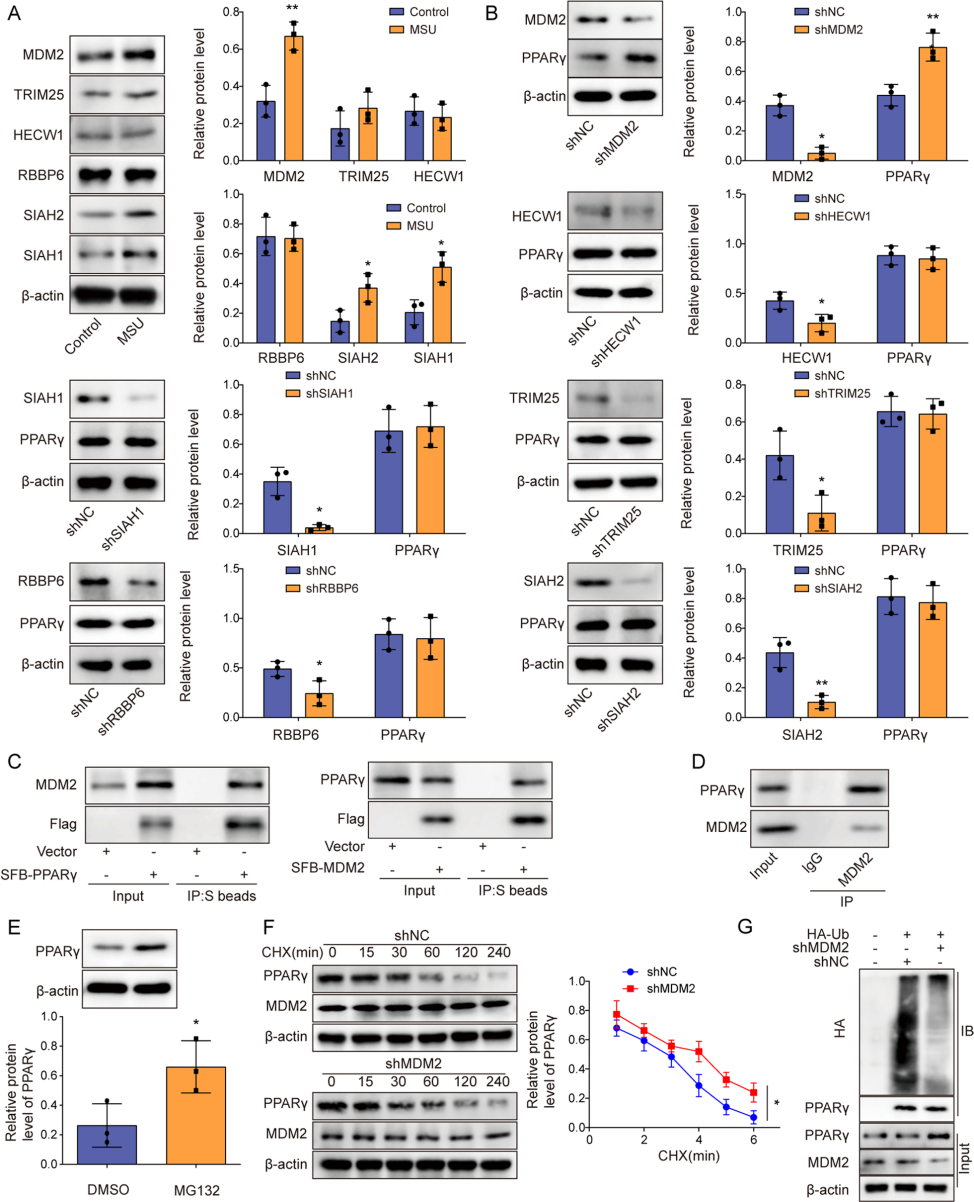
MDM2 reduced PPARγ protein stability via the ubiquitin–proteasome degradation pathway. **A** J774 cells were treated with LPS and MSU, after which the protein levels of MDM2, TRIM25, HECW1, RBBP6, SIAH2, and SIAH1 were determined through Western blotting. **B** PPARγ expression in J774 cells was assessed via Western blotting. **C**, **D** Co-IP assay was performed to analyze the interaction between exogenous/endogenous MDM2 and PPARγ in 293 T and J774 cells. **E** The expression of PPARγ was detected by Western blot after the addition of MG132. **F** The degradation of PPARγ was assessed by Western blot after the addition of CHX. **G** The ubiquitination-mediated degradation of PPARγ was detected by Co-IP. Measurement data are presented as mean ± SD. All data were obtained from three replicate experiments. *P < 0.05, **P < 0.01, ***P < 0.001

The mechanisms responsible for the downregulation of PPARγ protein expression in GA was subsequently investigated. Focusing on ubiquitination, the top six E3 ubiquitin ligases associated with PPARγ, including MDM2, TRIM25, HECW1, RBBP6, SIAH2, and SIAH1 in Mus musculus, were analyzed for their expression in GA cells using the UbiBrowser database. Our results demonstrated that MDM2, SIAH2, and SIAH1 expression was significantly increased in GA cells, whereas TRIM25, HECW1, and RBBP6 expression experienced little change in GA cells (Fig. 2A). Our results subsequently revealed that only MDM2 downregulation promoted an upregulation in PPARγ protein expression in GA cells, exhibiting a negative correlation (Fig. 2B). This supports the hypothesis that MDM2 may mediate PPARγ ubiquitination degradation. To validate our hypothesis, we utilized Co-IP assay to investigate potential protein–protein interactions. Our data unequivocally demonstrated that both exogenous and endogenous MDM2 directly interacted with PPARγ in J774 and 293 T cells (Fig. 2C, D). Meanwhile, we found that TRIM25, RBBP6, SIAH2, did not bind with PPARγ (Fig. S1A–C). To further determine the functional consequences of this interaction, we employed pharmacological intervention using MG132, a proteasome inhibitor. Notably, MG132 administration effectively elevated PPARγ protein levels (Fig. 2E). Complementary to this, MDM2 silencing markedly attenuated PPARγ protein degradation (Fig. 2F) and concomitantly inhibited PPARγ ubiquitination (Fig. 2G). In summary, MDM2 mediated PPARγ ubiquitination degradation by interacting with the PPARγ protein.

### MDM2 silencing inhibited NLRP3 activation and pyroptosis in GA cells by upregulating PPARγ protein expression

**Fig. 3:**
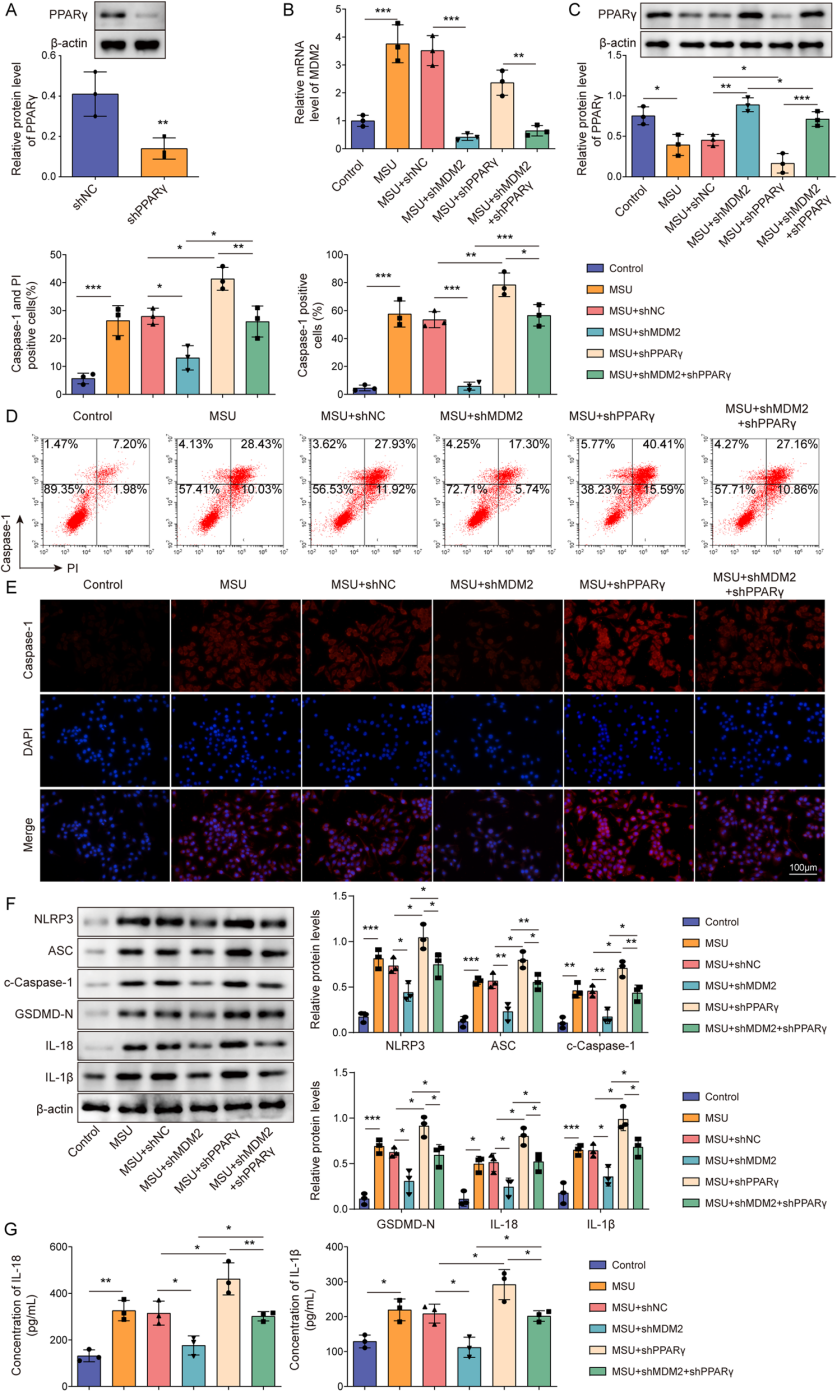
MDM2 silencing inhibited NLRP3 activation and pyroptosis in GA cells by upregulating PPARγ protein expression. **A** PPARγ expression in J774 cells after sh-NC or sh-PPARγ transfection was assessed though Western blotting assay. **B** MDM2 mRNA levels in J774 cells after sh-NC or sh-MDM2 transfection were measured using RT-qPCR. PPARγ knockdown and MDM2 knockdown were induced in LPS- and MSU-treated J774 cells. **C** PPARγ expression was assessed via Western blotting. **D** Flow cytometry following caspase-1/PI staining was employed to evaluate the state of cell pyroptosis. **E** Immunofluorescence was used to observe changes in c-caspase-1 (scale bar = 100 µm). **F** Western blotting was performed to assess the expression levels of proteins (NLRP3, ASC, c-caspase-1, GSDMD-N, IL-1β and IL-18). **G** ELISA was performed to determine IL-1β and IL-18 in cell supernatants. Measurement data are presented as mean ± SD. All data were obtained from three replicate experiments. *P < 0.05, **P < 0.01, ***P < 0.001

After further investigating the effects of MDM2-mediated PPARγ degradation, we found that MDM2 was upregulated in the GA cell model (Fig. 2A**)**. Subsequently, we constructed PPARγ-knockdown J774 cells (Fig. 3A). Moreover, in MDM2- and/or PPARγ-knockdown GA cells, we ascertained that PPARγ knockdown exacerbated LPS- and MSU-induced decrease in PPARγ protein levels, NLRP3 inflammasome activation, and pyroptosis in J774 cells, whereas MDM2 knockdown mitigated the effects of PPARγ knockdown (Fig. 3B–G). Furthermore, MDM2 silencing reversed PPARγ protein downregulation, NLRP3 inflammasome activation, and pyroptosis caused by PPARγ knockdown in GA cells (Fig. 3B–G). These findings demonstrated that MDM2 knockdown inhibited NLRP3 activation and pyroptosis in GA by upregulating PPARγ.

### BRD4 knockdown suppressed NLRP3 inflammasome activation and pyroptosis

**Fig. 4:**
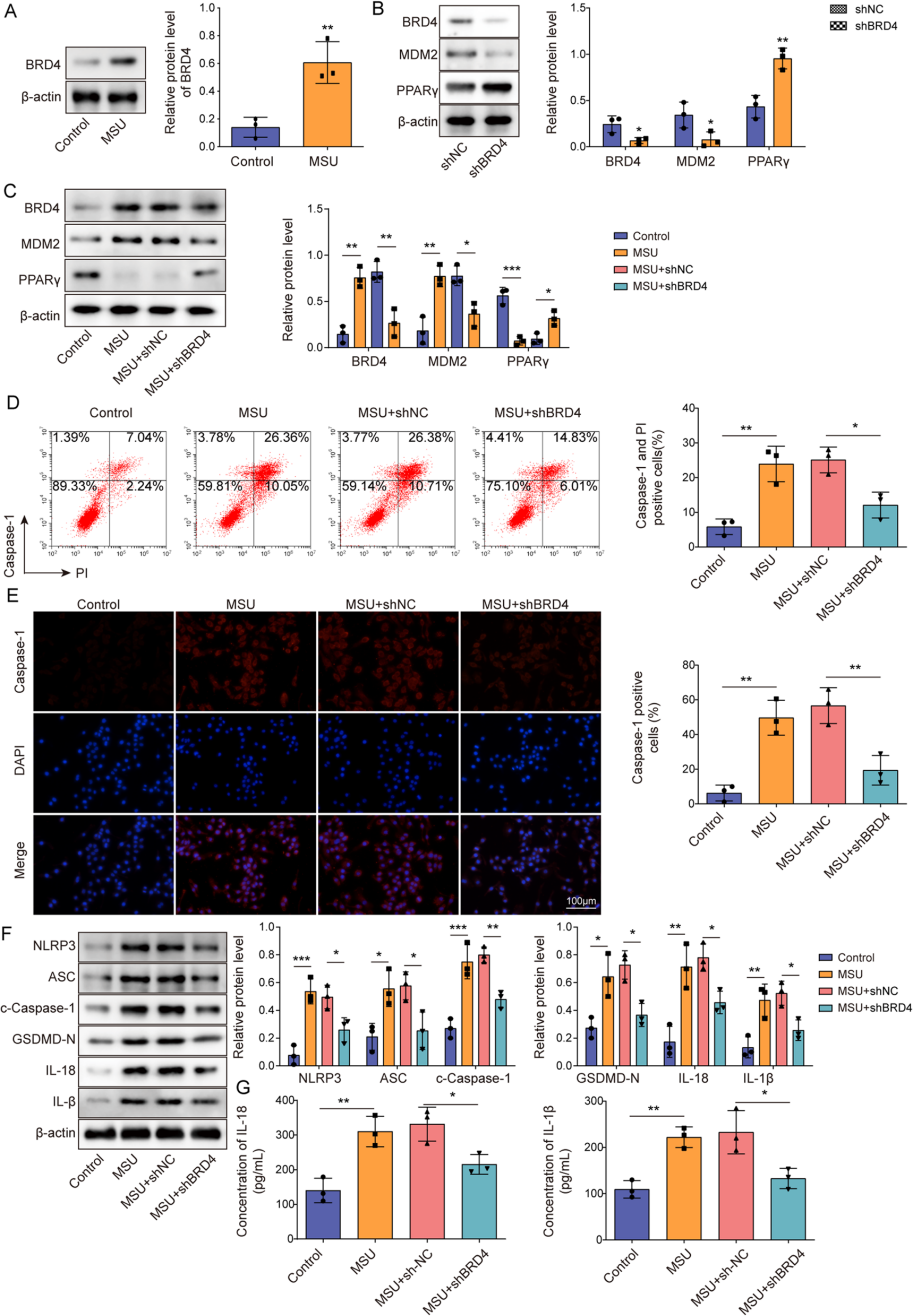
BRD4 knockdown suppressed NLRP3 inflammasome activation and pyroptosis. **A** J774 cells were treated with LPS and MSU, after which BRD4 protein levels in cells were examined via Western blotting. **B** BRD4, MDM2, and PPARγ protein levels in J774 cells following sh-NC or sh-BRD4 transfection were determined via Western blotting. J774 cells were treated with LPS and MSU and transfected with sh-NC or sh-BRD4. **C** Western blotting was utilized to measure BRD4, MDM2, and PPARγ protein levels in J774 cells. **D** Flow cytometry following caspase-1/PI staining was employed to assess the state of cellular pyroptosis. **E** Immunofluorescence was conducted to observe changes in c-caspase-1 levels (scale bar = 100 µm). **F** Western blotting was performed to evaluate the expression levels of proteins (NLRP3, ASC, c-caspase-1, GSDMD-N, IL-1β and IL-18). **G** ELISA assays were performed to determine IL-1β and IL-18 levels. *P < 0.05, **P < 0.01, ***P < 0.001. Measurement data are presented as mean ± SD. All data were obtained from three replicate experiments

Studies have found that the transcription factor BRD4 functioned as a known regulator of pyroptosis in GA (Hao et al. 2020). To determine the functional impact of BRD4 on inflammation and pyroptosis in GA, we utilized an in vitro model employing J774 cells subjected to LPS and MSU treatments and found BRD4 was highly expressed in GA cell model (Fig. 4A). Further study corroborated that sh-BRD4 transfection reduced BRD4 and MDM2 protein levels while boosting PPARγ levels in J774 cells (Fig. 4B). Moreover, BRD4 knockdown effectively reversed the promotive effects of LPS and MSU on BRD4 and MDM2 levels in J774 cells and their inhibitory effects on PPARγ level (Fig. 4C). Functionally, BRD4 knockdown mitigated LPS- and MSU-induced pyroptosis, including reducing the expression of NLRP3, ASC, GSDMD-N, and c-caspase-1 proteins and increasing the concentrations of IL-1β and IL-18 (Fig. 4D–G). Taken together, BRD4 knockdown inhibited NLRP3 inflammasome activation and pyroptosis in GA.

### BRD4 transcriptionally upregulated MDM2 expression

**Fig. 5:**
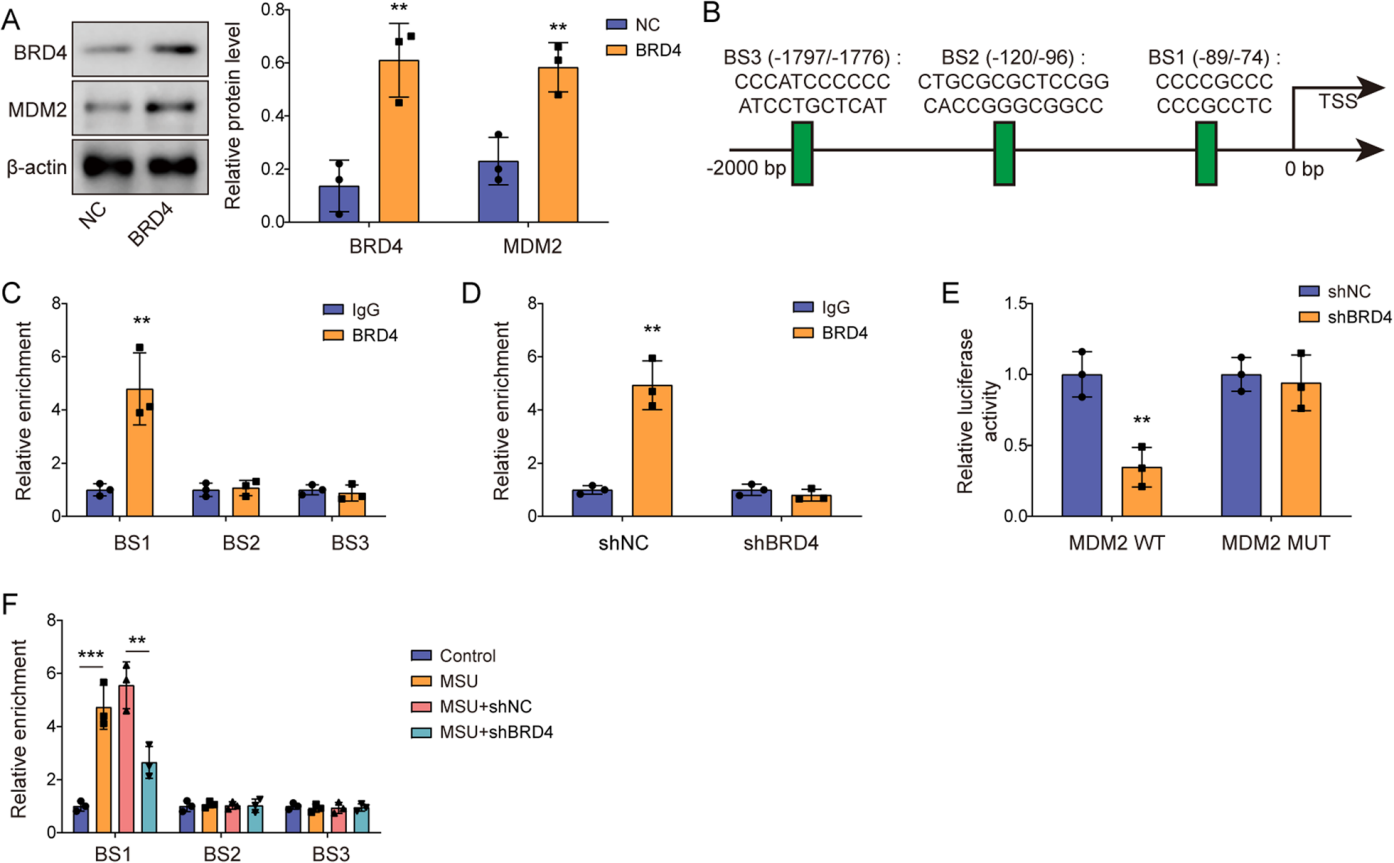
BRD4 transcriptionally upregulated MDM2 expression. **A** Western blotting was employed to assess BRD4 and MDM2 protein levels in J774 cells after NC or OE-BRD4 transfection. **B** The AnimalTFDB database was utilized to predict the interaction between BRD4 and the MDM2 promoter. **C** ChIP was performed to confirm the interaction between BRD4 and BS1–3 sites. **D** ChIP was used to detect BRD4 on the MDM2 promoter after BRD4 silencing. **E** Dual-luciferase assay measured the transcriptional activity of BRD4 on the MDM2 BS1 site. **F** ChIP was conducted to assess the binding of BRD4 to BS1–3 sites in the BRD4-knockdown GA cell model. Measurement data are presented as mean ± SD. All data were obtained from three replicate experiments. **P < 0.01, ***P < 0.001

The regulatory relationship between BRD4 and MDM2 during GA development was subsequently studied. BRD4 overexpression was induced in J774 cells by transfecting OE-BRD4 into cells. We subsequently observed that OE-BRD4 transfection significantly increased MDM2 levels in J774 cells (Fig. 5A). Hence, we predicted that BRD4 had potential binding sites on the MDM2 promoter using the AnimalTFDB database (Fig. 5B). To explore specific binding sites of BRD4 on MDM2, through the ChIP assay was conducted, targeting the three most likely binding sites of BRD4 on MDM2 (BS1–3, Fig. 5B). Our results showed that BRD4 overexpression significantly increased its enrichment on BS1 but not on BS2 or BS3 (Fig. 5C). To further validate this interaction, we observed that BRD4 knockdown lowered the enrichment of BRD4 on the MDM2 promoter (Fig. 5D). Moreover, BRD4 knockdown markedly decreased luciferase activity in MDM2-WT cells but not in BS1 mutant cells (Fig. 5E). Furthermore, BRD4 enrichment at BS1 was enhanced in LPS- and MSU- treated J774 cells, which was reversed by BRD4 silencing; however, no such effects were observed for BS2 and BS3 (Fig. 5F). These results conclusively identified BS1 as the specific binding site for BRD4. Collectively, BRD4 transcriptionally activated MDM2 expression by binding to the promoter region at BS1.

### BRD4 promoted NLRP3 inflammasome activation and pyroptosis via MDM2-mediated PPARγ degradation

**Fig. 6:**
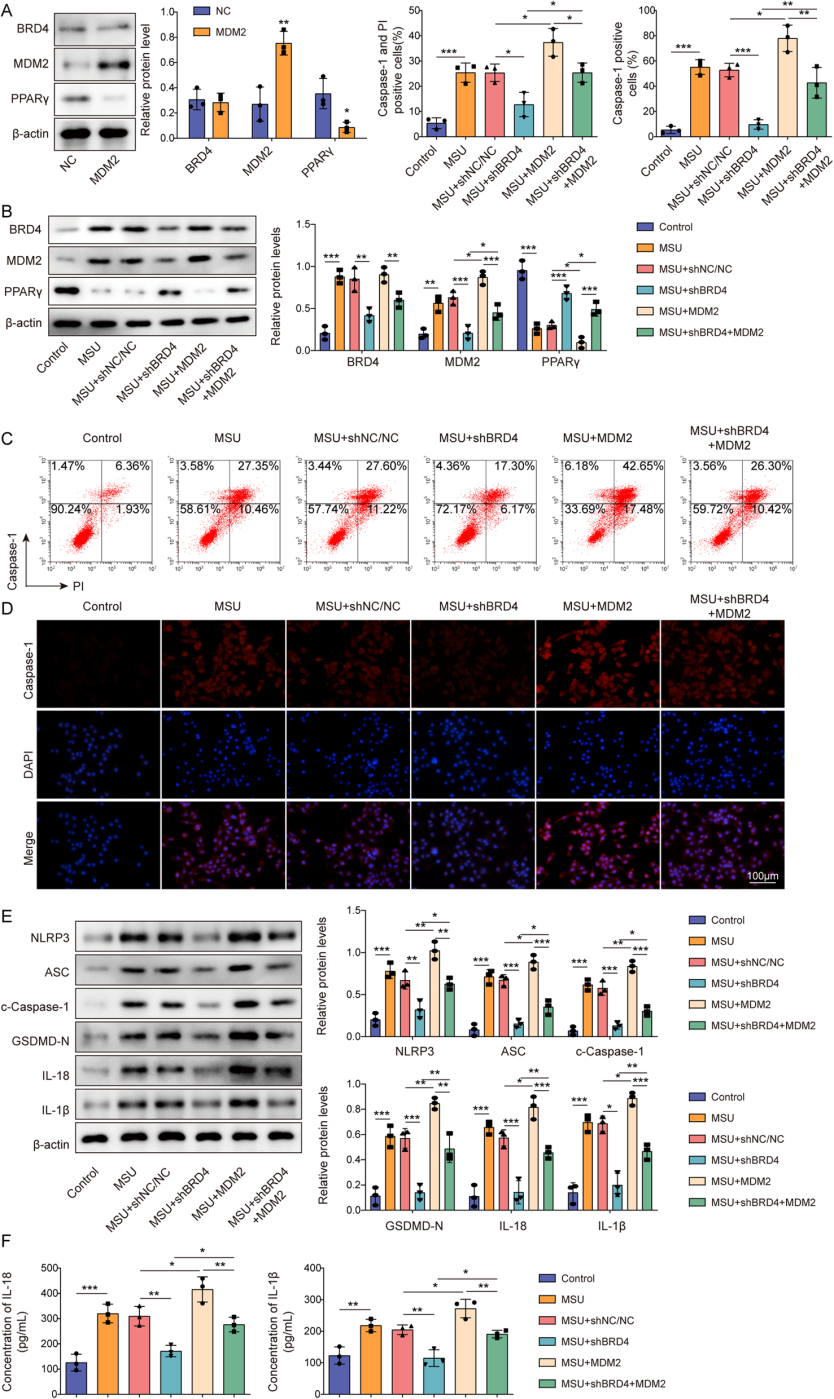
BRD4 promoted NLRP3 inflammasome activation and pyroptosis via MDM2-mediated PPARγ degradation. **A** Western blotting was utilized to measure the protein levels of BRD4, MDM2, and PPARγ in J774 cells following NC or OE-MDM2 transfection. Both BRD4 knockdown and MDM2 overexpression were induced in LPS- and MSU-treated J774 cells. **B** BRD4, MDM2, and PPARγ in J774 cells were examined through Western blotting. **C** Flow cytometry following caspase-1/PI staining was employed to assess the state of cellular pyroptosis. **D** Immunofluorescence was conducted to observe changes in c-caspase-1 levels (scale bar = 100 µm). **E** Western blotting was conducted to evaluate the expression leves of pyroptosis-related proteins. **F** ELISA assays were performed to determine IL-1β and IL-18 levels. Measurement data are presented as mean ± SD. All data were obtained from three replicate experiments. *P < 0.05, **P < 0.01, ***P < 0.001

The functional impact of the BRD4–MDM2–PPARγ axis on inflammation and pyroptosis in GA was studied. Notably, we found that transfecting OE-MDM2 into J774 cells induced MDM2 overexpression and that OE-MDM2 transfection significantly increased MDM2 protein level and reduced PPARγ level in cells (Fig. 6A). After constructing an in vitro model employing BRD4-knockdown and/or MDM2-overexpressed J774 cells subjected to LPS and MSU treatments, we found that BRD4 and MDM2 were upregulated and that PPARγ was downregulated in the GA cell model. Further study revealed that BRD4 knockdown reduced BRD4 and MDM2 levels but boosted PPARγ levels, whereas MDM2 overexpression demonstrated the opposite trend toward MDM2 and PPARγ levels and had no impact on BRD4 levels. Moreover, BRD4 knockdown was able to reversed the increase in MDM2 levels and decrease in PPARγ levels induced by MDM2 overexpression (Fig. 6B). Functionally, BRD4 knockdown mitigated LPS- and MSU-induced NLRP3 inflammasome activation and pyroptotic cell death, with such effects being amplified by MDM2 overexpression. Conversely, the ameliorative impact of BRD4 knockdown on NLRP3 activation and pyroptosis was reversed upon simultaneous MDM2 overexpression (Fig. 6C–F). Collectively, these findings substantiate the hypothesis that BRD4 modulates NLRP3 inflammasome activation and pyroptosis in GA through an MDM2-mediated PPARγ degradation pathway.

### BRD4 silencing improved GA in vivo through MDM2-mediated PPARγ degradation-regulated pyroptosis

**Fig. 7:**
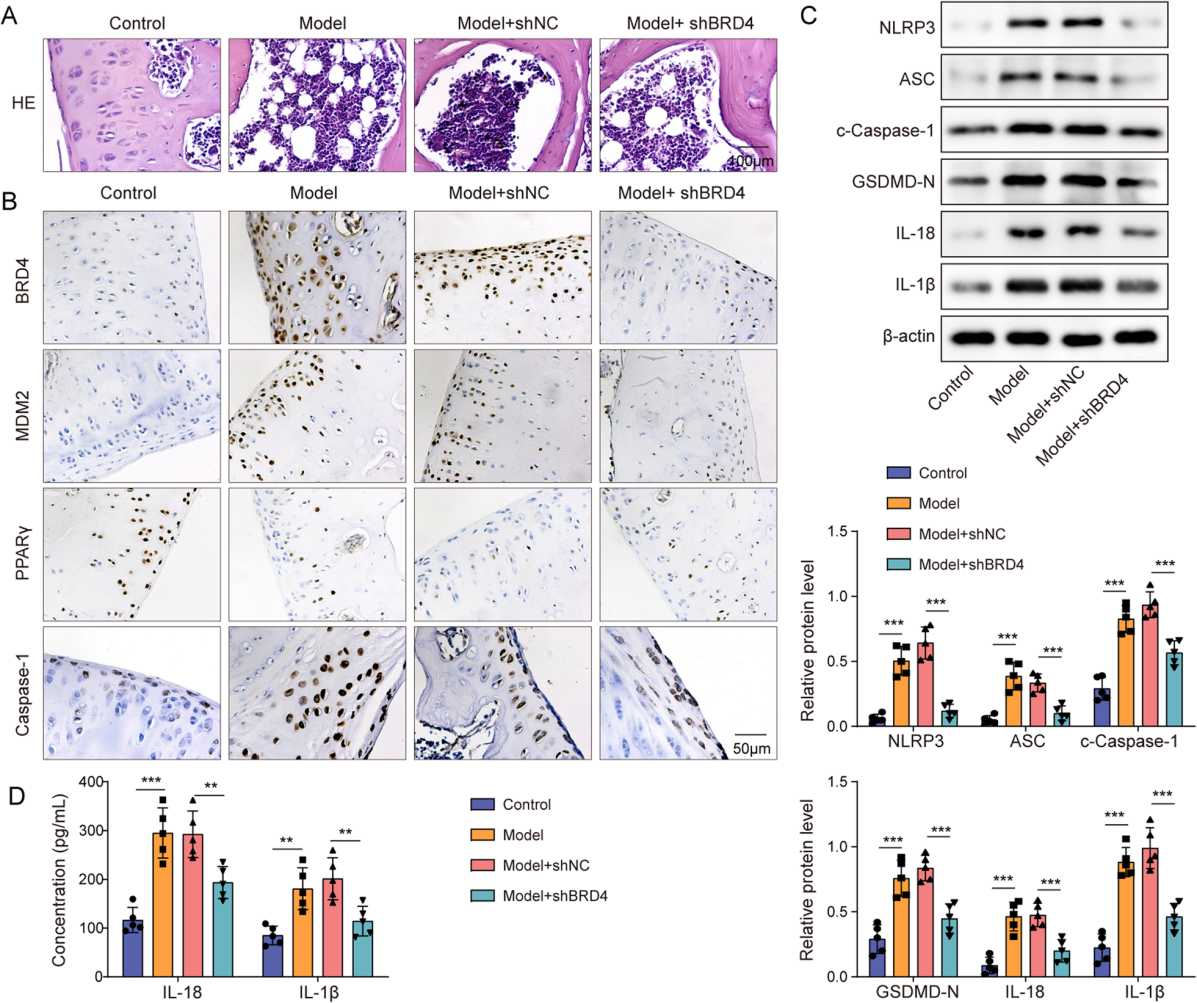
BRD4 silencing improved GA in vivo through MDM2-mediated PPARγ degradation-regulated pyroptosis. We established a GA animal model by injecting MSU crystals into the joint of C57BL/6 J mice. Concurrently, sh-BRD4 vectors were locally administered. **A** H&E staining of representative knee joint synovial tissue sections. (Scale bar = 100 µm). **B** Immunohistochemistry was employed to assess tissue protein expression. (Scale bar = 50 µm). **C** Western blotting was performed to evaluate theexpression levels of pyroptosis-related proteins. **D** ELISA assays were conducted to evaluate IL-1β and IL-18 levels. Measurement data are presented as mean ± SD. N = 5. **P < 0.01, ***P < 0.001

To determine the role of BRD4 and PPARγ in GA in vivo, MSU crystals were injected into the joint of C57BL/6 J mice to establish an animal model of GA. The animals subsequently received a local administration of sh-BRD4 vector. Histopathological analyses revealed that MSU-induced synovial thickening, chondrocyte vacuolation, inflammatory cell infiltration, interstitial swelling, and exudate formation in the joint tissue, whereas BRD4 silencing mitigated these pathological changes (Fig. 7A). Molecular analyses further revealed that MSU treatment elevated BRD4 and MDM2 expression while suppressing PPARγ levels, which were counteracted by BRD4 knockdown (Fig. 7B). Moreover, elevated levels of NLRP3, ASC, GSDMD-N, c-caspase-1, IL-1β, and IL-18 were observed following MSU treatment but were reversed upon BRD4 silencing (Fig. 7B–D). Combining these results, we ascertained that BRD4 inhibition attenuates GA symptoms and NLRP3 inflammasome-mediated pyroptosis mechanistically via the MDM2-mediated PPARγ degradation pathway.

## Discussion

The present study investigated the complex mechanisms governing pyroptosis in the context of GA. Specifically, we revealed that BRD4 promoted GA development by regulating MDM2-mediated PPARγ degradation and pyroptosis. Mechanistically, BRD4 promoted MDM2-mediated PPARγ degradation and pyroptosis by transcriptionally activating MDM2 expression.

BRD4, a critical member of the bromodomain and extraterminal domain (BET) family, plays pivotal roles in gene expression, cell cycle progression, and chromatin remodeling. Its role in regulating gene expression and cellular pathways makes it a promising target for diverse therapeutic interventions. Our research underscores the therapeutic utility of BRD4 inhibition in the management of GA, with particular emphasis on its modulatory effects on inflammation and pyroptosis. BRD4 silencing substantially ameliorated GA symptoms and mitigated the activation of inflammatory pathways, specifically mediating pyroptosis. Notably, our current experiments were performed in J774 cells. One previous study showed that RAW264.7 cells lacks ASC (Bryan et al. 2010). However, previous studies have detected ASC in RAW264.7 cells, indicating that RAW264.7 cells expressed ASC (Zhang et al. 2018; Sun et al. 2019; Xie et al. 2014). To avoid controversy, we replaced RAW264.7 with J774 cells and repeated all experiments. Consistent with our findings, Kun et al. substantiated the preventative potential of BRD4 inhibition in GA by regulating pyroptosis (Hao et al. 2020). They observed that both the BRD4 inhibitor JQ1 and gene silencing techniques significantly alleviated MSU-induced pyroptosis and joint inflammation in murine models. Similarly, Shao et al. identified BRD4 upregulation in GA, which could be attributed to the inflammation resulting from microRNA-mediated regulation of BRD4 (Shao et al. 2023). In addition, BRD4 inhibitor treatment was able to remarkably alleviate GA symptoms (Jiang et al. 2019). Collectively, these findings solidify the position of BRD4 as a viable therapeutic target for GA and suggest its broader applicability in treating associated inflammatory diseases.

BRD4 has been acknowledged as an epigenetic reader, recognizing acetylated histones and facilitating the recruitment of the RNA polymerase II complex and other transcriptional machinery to specific genomic locations (Gong et al. 2022; Dey et al. 2019; Wang et al. 2021). Our study provides additional insights into the role of BRD4, demonstrating its capacity to modulate pyroptosis via transcriptional activation of MDM2 in GA. This initiates a molecular cascade that culminates in the ubiquitination and degradation of PPARγ. Although no study has yet clearly described the mechanism by which BRD4 transcriptionally controls MDM2, Hines et al. found that MDM2 promoted BRD4 degradation via the ubiquitin–proteasome pathway (Hines et al. 2019). These findings indicate an intricate regulatory interplay between BRD4 and MDM2, highlighting the need for future studies on their reciprocal regulation in the pathophysiology of GA.

MDM2 is a well-characterized E3 ubiquitin ligase renowned for its pivotal role in modulating the cellular levels of the tumor suppressor p53 (Chinnam et al. 2022). Aside from its interaction with p53, MDM2 interacts with a wide range of other cellular proteins and is involved in various cell processes, including cell cycle regulation (Giono et al. 2017), autophagy (Kook et al. 2020), and apoptosis (Foo et al. 2007). The current study revealed that MDM2 served as a key mediator in regulating pyroptosis and inflammation during the progression of GA. This regulation occurs through mechanisms involving the ubiquitination and subsequent degradation of PPARγ. Corroborating our findings, a previous study showed that MDM2 regulated the ubiquitination of PPARγ (Cai et al. 2022). Furthermore, Xu et al. found that MDM2 binds to PPARγ and is responsible for its ubiquitin-mediated degradation, emphasizing that this process requires the involvement of EGFR (Xu et al. 2016). Shi et al. subsequently confirmed that inhibiting the EGFR/MDM2 pathway significantly curtails PPARγ degradation (Shi et al. 2016). Notably, Hallenborg et al. discovered that MDM2 indirectly modulates PPARγ expression by mediating the nuclear sequestration of transcriptional cofactors MORC2 and LIPIN1 in adipose cells (Hallenborg et al. 2021). These findings suggest an intricate mechanism through which MDM2 regulates PPARγ expression, warranting further investigation in future studies.

PPARγ can transcriptionally regulate the expression of genes involved in lipid metabolism, glucose homeostasis, and inflammation (Gao et al. 2015; Han et al. 2017; Hernandez-Quiles et al. 2021). It has been increasingly recognized for its versatile roles in immune response modulation, extending its importance beyond metabolic disorders to include a broader range of pathophysiological conditions. Our study identified PPARγ as an active participant in the pathology of GA. We found that MDM2-mediated degradation of PPARγ significantly accelerates the progression of GA. In support of our findings, Wang et al. reported that PPARγ activation effectively ameliorated GA symptoms by modulating inflammatory factors such as IL-1β (Wang et al. 2019). Similarly, another study demonstrated that inducers of PPARγ improved GA by regulating inflammatory factors (Wang and Jiang 2014a). Du et al. further corroborated these findings by verifying that PPARγ activation attenuates GA by mitigating NLRP3-associated inflammation (Du et al. 2023). Beyond these insights, we reported that PPARγ degradation serves as a catalyst for inflammation and pyroptosis in GA. Although considerable research has verified the association between PPARγ and pyroptosis in other conditions, including ulcerative colitis (Wang et al. 2022), sepsis (Li et al. 2022) and osteoarthritis (Feng et al. 2023), only a few studies have investigated this relationship in the context of GA. Our study expands the mechanistic understanding of PPARγ in GA and highlights avenues for future research focused on PPARγ as a potential therapeutic target for managing GA.

## Conclusions

The currents study revealed that BRD4 can promote GA by regulating pyroptosis. Mechanistically, BRD4 transcriptionally activates MDM2, which consequently leads to the ubiquitination and degradation of PPARγ, thereby inducing pyroptosis and inflammation.

### Supplementary Information

Supplementary Material 1: Figure S1. Interactions between TRIM25, RBBP6, SIAH2, and PPARγ were analyzed via the Co-IP assay. (A) Interaction between TRIM25 and PPARγ was analyzed via the Co-IP assay. (B) Interaction between RBBP6 and PPARγ was analyzed via the Co-IP assay. (C) Interaction between SIAH2 and PPARγ was analyzed via the Co-IP assay.

## Data Availability

Data sharing not applicable to this article as no datasets were generated or analysed during the current study.

## Abbreviations

BET: Bromodomain and extraterminal domain
BRD4: Bromodomain-containing protein 4
ChIP: Chromatin immunoprecipitation
CHX: Cycloheximide
Co-IP: Co-immunoprecipitation
DMEM: Dulbecco’s Modified Eagle Medium
ELISA: Enzyme-linked immunosorbent assay
FBS: Fetal bovine serum
GA: Gouty arthritis
H&E: Hematoxylin and eosin
IHC: Immunohistochemical
LPS: Lipopolysaccharide
MDM2: Mouse double minute 2 homolog
MSU: Monosodium urate
PPARγ: Peroxisome proliferator-activated receptor gamma
PTM: Post-transcriptional modulatory mechanisms
RT-qPCR: Real-time quantitative PCR
SD: Standard deviation
