# Supplementary figures and images for: BRD4 promotes gouty arthritis through MDM2-mediated PPARγ degradation and pyroptosis
Source: Mol Med. 2024 May 21;30:67. doi: 10.1186/s10020-024-00831-w (PMC11110350; doi:10.1186/s10020-024-00831-w)

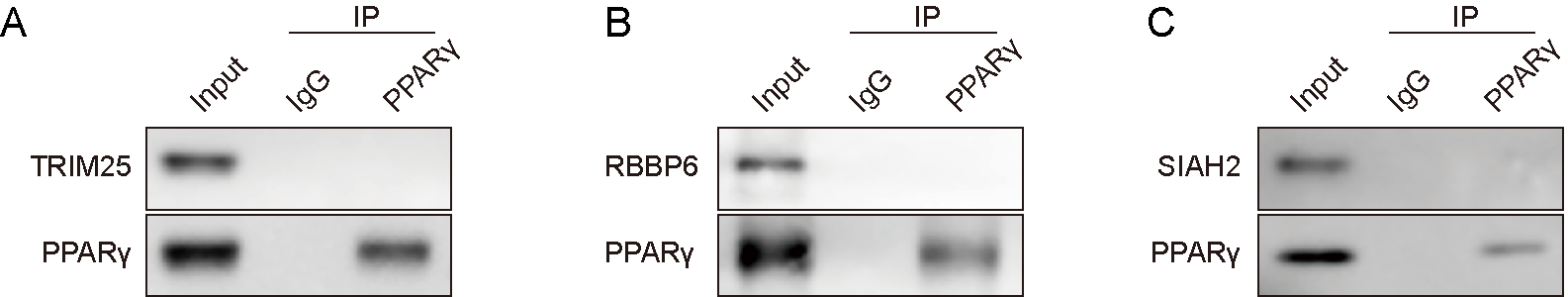

Supplement: Supplementary file 1 — Supplementary Material 1: Figure S1. Interactions between TRIM25, RBBP6, SIAH2, and PPARγ were analyzed via the Co-IP assay. (A) Interaction between TRIM25 and PPARγ was analyzed via the Co-IP assay. (B) Interaction between RBBP6 and PPARγ was analyzed via the Co-IP assay. (C) Interaction between SIAH2 and PPARγ was analyzed via the Co-IP assay. [file 10020_2024_831_MOESM1_ESM.tif]
